# Antifibrinolytics in liver surgery

**DOI:** 10.4103/0019-5049.72636

**Published:** 2010

**Authors:** Jalpa Makwana, Saloni Paranjape, Jyotsna Goswami

**Affiliations:** Department of Anaesthesia, Jaslok Hospital and Research Centre, Mumbai, India

**Keywords:** Antifibrinolytic drugs, blood transfusion, hyperfibrinolysis, orthotopic liver transplantation

## Abstract

Hyperfibrinolysis, a known complication of liver surgery and orthotopic liver transplantation (OLT), plays a significant role in blood loss. This fact justifies the use of antifibrinolytic drugs during these procedures. Two groups of drug namely lysine analogues [epsilon aminocaproic acid (EACA) and tranexamic acid (TA)] and serine-protease-inhibitors (aprotinin) are frequently used for this purpose. But uniform data or guidelines on the type of antifibrinolytic drugs to be used, their indications and correct dose, is still insufficient. Antifibrinolytics behave like a double-edged sword. On one hand, there are benefits of less transfusion requirements but on the other hand there is potential complication like thromboembolism, which has been reported in several studies. We performed a systematic search in PubMed and Cochrane Library, and we included studies wherein antifibrinolytic drugs (EACA, TA, or aprotinin) were compared with each other or with controls/placebo. We analysed factors like intraoperative red blood cell and fresh frozen plasma requirements, the perioperative incidence of hepatic artery thrombosis, venous thromboembolic events and mortality. Among the three drugs, EACA is least studied. Use of extensively studied drug like aprotinin has been restricted because of its side effects. Haemostatic effect of aprotinin and tranexamic acid has been comparable. However, proper patient selection and individualized treatment for each of them is required. Purpose of this review is to study various clinical trials on antifibrinolytic drugs and address the related issues like benefits claimed and associated potential complications.

## INTRODUCTION

Major blood loss is a known complication in liver resection and liver transplantation, with a multi-factorial origin. Hyper-fibrinolysis plays a significant role in non-surgical blood loss requiring massive transfusion. Red blood cell (RBC) and platelet transfusions are independent risk factors for adverse outcomes after liver transplantation.[1] Primary hyper-fibrinolysis that occurs during liver surgery is the basis for the use of antifibrinolytic agents to reduce blood loss and transfusion requirements. Two groups of antifibrinolytics are available: lysine analogues (epsilon aminocaproic acid and tranexamic acid) and serine protease inhibitors (aprotinin). Of these drugs, aprotinin has been the most extensively studied but is now in disrepute as it is reported to increase mortality in cardiac surgery. Tranexamic acid is more commonly used and found to be effective in decreasing transfusion requirements.

## SEARCH STRATEGY AND DATA ANALYSIS

A systemic literature search was conducted in PubMed and the Cochrane Library from 1966 till date. The search strategy was set up using the following single text words and combinations: aprotinin, ε-aminocaproic acid (EACA), tranexamic acid (TA), antifibrinolytic drug, antifibrinolytics and liver transplantation. Reference lists of relevant articles were cross checked for other potentially relevant articles.

In the systematic review all trials, both randomized and non-randomized, comparing antifibrinolytic drugs among each other or with placebo/controls were included.

The following data were considered. Red blood cell (RBC) and fresh frozen plasma (FFP) transfusion requirements during transplantation, perioperative hepatic artery thrombosis and venous thromboembolic events. We also compared the various drugs (TA, EACA, aprotinin), irrespective of the dosage used.

## HAEMOSTATIC CHANGES DURING LIVER TRANSPLANTATION

During the anhepatic phase, circulating levels of plasminogen activator inhibitor (PAI), which is synthesized by the liver, are reduced leading to increase in tissue plasminogen activator (t-PA). t-PA is the major activator for the conversion of plasminogen to plasmin resulting in fibrinolysis. At reperfusion, there is an unpredictable but accelerated release of t-PA from the graft endothelium which causes generalized fibrinolysis and surgical bleeding.[2–5]

## HAEMOSTATIC CHANGES DURING LIVER RESECTION

There may be a variable degree of hyperfibrinolytic state during liver resection. This event is more pronounced in patients with diseased liver or who undergo wider hepatectomy. The hyperfbrinolytic state develops immediately after liver resection with peak effect on the 1^st^ postoperative day and returns to normal only after 3 -7 days.[6]

## ANTIFIBRINOLYTIC AGENTS

Two groups of drugs are used to inhibit fibrinolysis: lysine analogues (epsilon aminocaproic acid and tranexamic acid) and the serine protease inhibitor (aprotinin).

### Epsilon aminocaproic acid

Epsilon aminocaproic acid (EACA) is a synthetic lysine analogue. It binds reversibly to the kringle domain of the enzyme plasminogen, and competitively inhibits the binding of plasminogen to lysine residue on the surface of fibrin and prevents conversion of plasminogen to plasmin. Some studies have shown that it also inhibits pro-urokinase-induced plasminogen activation and prevents plasmin degradation of platelet glycoprotein Ib receptors, thus preserving platelet function.[7,8] It is primarily metabolised and eliminated by kidney. Sixty-five percent of drug is found unchanged in urine. Its half-life is about 2 hours. EACA is associated with renal complications. Acute renal failure may be due to Acute tubular necrosis (ATN), renal infarction, myopathy, pigment-induced renal complications, glomerular capillary thrombosis and elevated excretion of beta-2 microglobulin.

### Tranexamic acid

A 4-aminomethyl cyclohexane-carbolic acid, which is a synthetic derivative of the amino acid lysine. Tranexamic acid (TA) prevents plasmin-mediated conversion of fibrinogen to fibrinogen split products by competitively binding to the lysine binding sites on the plasminogen molecule. It also inhibits the action of plasminogen and plasmin on platelets and exerts a protective effect on platelets. At higher concentrations, TA may also act as non-competitive inhibitor of plasmin.[9] It is 6–10 times more potent than EACA and has a longer half-life, which is about 3.1 hours. As compared to EACA, its antifibrinolytic activity is higher in peripheral compartments like kidney, intestines, and prostatic tissues.[10] Kidney is the primary organ for its excretion where about 95% of drug is eliminated in unchanged form. TA is a well-tolerated drug with less adverse effects. However, it is reported to be associated with nausea, vomiting, diarrhoea, orthostatic reactions and retinal changes.

### Aprotinin

It is a naturally occurring protease inhibitor derived from bovine and porcine lung. It inhibits various proteases like human plasmin, trypsin, kallikrein, chymotrypsin, activated protein C and thrombin.[11,12] It forms an aprotinin-enzyme complex on the active serine site of the enzyme which has a specific dissociation constant for aprotinin – highest with trypsin, moderate with plasmin and lowest with kallikrein. Inhibition of kallikrein requires a higher dose of aprotinin than inhibition of plasmin. Mechanism of action of aprotinin is complex including inhibition of plasmin, contact activation system (via kallikrein inhibition) and tissue-plasminogen activator production. In addition to antifibrinolytic effect, aprotinin also has antithrombotic effects, which may be due to selective blockade of proteolytically activated thrombin receptors (PAR1) on platelets.[13] The proteases are a part of various inflammatory cascades, which may explain the role of aprotinin in decreasing the inflammatory response during major surgery. The terminal half-life is 7–8 hours. There have been concerns about the safety of aprotinin. Side effects like anaphylaxis and thrombosis could lead to renal failure, myocardial infarction, heart failure, stroke and encephalopathy. Bayer withdrew aprotinin in November 2007 because Fergussion and others[14] showed an increased risk of death when used to prevent bleeding during cardiac surgery. The study compared aprotinin with lysine analogues (TA and EACA) in patients undergoing cardiac surgery. Although there was less bleeding with aprotinin, the trial was prematurely terminated because of higher death rates in patients receiving aprotinin. The FDA (USA) now recommends restricted use of aprotinin only in patients with increased risk of bleeding when no other acceptable alternative is available and that the physician using aprotinin in such a situation must outweigh the risk for the patient.[15]

### Review of literature

#### EACA

EACA was first used in liver transplantation in 1966.[16] In a study of 97 patients undergoing OLT, 20 patients developed severe hyperfibrinolytic state and were treated successfully with 1 gm of EACA.[3] In another randomized placebo-controlled study using EACA 16 mg/kg/h, TA 10 mg/kg/h and placebo, EACA reduced RBC and FFP transfusion requirement, but this was not statistically significant, whereas TA significantly decreased fibrinolysis and intraoperative RBC requirements. The incidence of thrombosis did not differ among the 3 groups.[17]

#### TA

Use of TA in OLT was first reported in the 1980s.[18] Later on TA was compared with placebo in 45 patients of OLT, where TA (20 mg/kg) showed significantly less intraoperative blood loss and reduced transfusion requirements.[19] No patient had hepatic artery or portal vein thrombosis. But other investigators failed to demonstrate efficacy of small dose of TA (2 mg/kg/h) in reducing transfusion requirements and reported one case of hepatic artery thrombosis postoperatively with TA.[4] In another randomized controlled study with TA (10 mg/kg/h) and aprotinin (2 million KIU bolus followed by 500,000 KIU/h infusions), Dalmau and others. did not find any significant difference in blood loss, transfusion requirements or perioperative complications.[20]

Wu and others conducted a prospective randomized trial to examine the feasibility of a blood transfusion-free hepatectomy. They administered TA 500 mg before surgery followed by 250 mg 6 hourly for 3 days, and observed significantly less intraoperative blood loss, lower transfusion rate and shorter operative time.[6]

#### Aprotinin

Neuhaus and others[21] first reported the clinical use of aprotinin in OLT in 1989 with a dose of 2 million Kallikren inhibition unit (KIU), which reduced blood loss, transfusion requirements and duration of surgery. Subsequently, several other reports supported this finding.[22–27] A comparative study with two different doses of aprotinin (high dose, i.e., 2 million KIU followed by an infusion of 500,000 KIU/h vs. low dose, i.e., 500,000 KIU followed by infusion of 150,000 KIU/h) found no significant difference in the rate of red cell transfusion between the high and low dose groups.[28] Another study showed that low dose aprotinin decreased cryoprecipitate and FFP requirements but not PRBC and platelet requirement[24] Garcia-Huete and others challenged its efficacy in a prospective trial comparing aprotinin (2×10^6^ KIU at induction followed by 5×10^6^ KIU/h infusions) with placebo and found similar intraoperative requirements of RBCs, Fresh frozen plasma (FFP), platelets and cryoprecipitate in both groups.[29]

Aprotinin also has anti-inflammatory and antioxidant effects, which helps to provide significantly better haemodynamic stability and a lesser degree of reperfusion syndrome in OLT.[30] The European Multicentre Study of Aprotinin in Liver transplant (EMSALT) showed a decrease in red blood cell usage with both high dose and regular dose of aprotinin.[26]

Aprotinin was also showed useful to reduce intraoperative blood loss and transfusion requirement in elective liver resection without any venous thrombosis.[31] Lentschener and others[32] reviewed the use of aprotinin in liver transplantation and concluded that prophylactic use of large dose aprotinin decreases blood loss and transfusion requirement only when OLT is associated with significant blood loss and did not alter postoperative outcome.

There are many reviews on the use of antifibrinolytics in liver surgery.[33,34] In one review; aprotinin appears to be more effective than TA and EACA. But authors commented that it has been studied more extensively.[33] Molenaar and others[34] reviewed antifibrinolytics in liver transplantation and concluded that both aprotinin and TA significantly reduce RBC transfusion requirements. Aprotinin, but not TA, significantly reduces the intra-operative use of FFP. There was no evidence of an increased risk of hepatic artery thrombosis, venous thromboembolic events or mortality in patients who received antifibrinolytics.

In a review evaluating haemostatic effect of aprotinin with nafamostat mesilate, improvements in surgical technique and anaesthesiological care were found to be more important in reducing blood loss than the use of the antifibrinolytic drugs in partial hepatectomy. In liver transplantation, aprotinin reduced blood loss and transfusion requirements by 30-40%. They concluded that scientific support for the routine use of aprotinin or nafamostat mesilate in partial hepatectomy was insufficient, whereas the efficacy of aprotinin in liver transplantation was confirmed.[35] However, pharmacological measures to reduce bleeding and transfusion requirements must be based on clinical evidence.[36]

Gurusamy and others[37] reviewed pharmacological interventions to decrease bleeding in liver resection using aprotinin, desmopressin, recombinant factor VIIa, antithrombin III and TA. There was no significant difference in the perioperative mortality, survival at maximal follow-up, liver failure or other perioperative morbidity. Transfusion requirement was significantly lower in the aprotinin and tranexamic acid groups. Authors concluded that there was high risk of type I and type II statistical errors because of few trials, the small sample size in each trial and due to high risk of bias.

## COMPLICATIONS

Thromboembolic phenomena are the most undesirable complications during liver transplantation manifesting as hepatic artery thrombosis, venous thromboembolism pulmonary thromboembolism. The pathogenesis of thromboembolism during OLT is complex. Several factors, which are inherent to the procedure of transplantation, can activate the coagulation system. Injury of a large capillary bed, venous stasis due to clamping (total or partial) of venacava or portal vein, ischemic insult of the intestine, activators released from the graft, massive blood loss, septic complications and use of venovenous bypass may all contribute to this increased risk for thromboembolic events. However, development of full-blown disseminated intravascular coagulation (DIC) and a consumption coagulopathy is rare.[38] In a recent review of thromboembolic complications in OLT identified seventy-four cases of intraoperative pulmonary embolism (PE) and/or intra cardiac thrombosis (ICT) during OLT; PE alone in 32 patients (43%) and a combination of PE and ICT in 42 patients (57%). PE and ICT occurred in every stage of the operation and were reported equally in patients with or without the use of venovenous bypass or antifibrinolytics. The authors commented that intraoperative PE and ICT during OLT have multiple aetiologies and may occur unexpectedly at any time during the procedure.[39]

Hepatic artery thrombosis is a serious complication, resulting in bile duct necrosis requiring re-transplantation. In a systematic review and meta-analysis, the incidence of hepatic artery thrombosis in placebo group was 2.5%, 4.6% with TA, 4.8% with EACA and 1.3% with aprotinin, which is lower than any other group.[34]

Venous thromboembolism is another concern, which has been reported by several authors.[3,40] There are at least 30 case reports of intraoperative thromboembolism. One case report did not mention the use of antifibrinolytics. In 5 out of 29 cases, antifibrinolytics were not used. Out of 24 patients receiving antifibrinolytics, 11 received aprotinin, 10 received EACA and 3 patients had received both EACA and aprotinin. No case of intraoperative thromboembolism was reported with TA.

In a recent review,[34] the incidence of venous thromboembolic events with TA was 0.7% (2/306, both postoperatively), with aprotinin 1.4% (5/349, 3 postoperative) and with placebo 1.5%.

Different studies are shown in tabular form [Table 1] with different antifibrinolytic agents, time of their administration, and their outcome.[38,41–51] Post-transplant morbidity in the form of thromboembolic complications differs in the recipients of live donor liver transplant (LDLT) and deceased donor liver transplant (DDLT)/ split liver transplant. In a retrospective cohort study, incidence of hepatic artery thrombosis was higher in LDLT (6.5%) than DDLT (2.3%), while portal vein thrombosis was 2.9% in LDLT and 0% in DDLT.[52] In a single centre study with 224 patients, the incidence of vascular complications was significantly higher with LDLT compared to DDLT (hepatic artery thrombosis- 4.3% vs. 3.2%, portal vein thrombosis- 7.2% vs. 2.6%, respectively).[53] However, a recent systematic review reported no difference in the incidence of early hepatic artery thrombosis between on LDLT (3.1%) and DDLT (4.6%, *P*=0.1).[54]

**Table 1:** Venous thromboembolism: Review of literature

Year	Author	Cases	Antifibrinolytic	Phase	Outcome
1988	Navalgund *et al*.	1	None	Anhepatic	Death
1994	Baubillier *et al*.	1	Aprotinin	Preanhepatic	Death
1995	Prah *et al*.	1	EACA	Preanhepatic	Survived
1995	Prah *et al*.	1	EACA	Preanhepatic	Death
1997	Sopher *et al*.	1	Aprotinin	Anhepatic	Survived
1997	Sopher *et al*.	1	EACA+Apro	Reperfusion	Death
1998	Manji *et al*.	1	Aprotinin	Reperfusion	Survived
2001	Gologorsky *et al*.	2	None	Reperfusion	Survived
2001	Gologorsky *et al*.	5	EACA	Reperfusion	Survived
2001	Fitzsimmons *et al*.	1	Aprotinin	Anhepatic	Survived
2001	Fitzsimmons *et al*.	1	Aprotinin	Anhepatic	Death
2002	O’connor *et al*.	1	Aprotinin	Dissection	Survived
2002	O’connor *et al*.	1	EACA+Apro	Anhepatic	Survived
2002	Wong *et al*.	2	EACA	Anhepatic	Survived
2004	Planinsic *et al*.	1	None	Preanhepatic	Survived
2004	Ramsay *et al*.	1	Aprotinin	Reperfusion	Death
2005	Lerner *et al*.	2	None	Preanhepatic anhepatic	Survived
2005	Lerner *et al*.	2	None	Anhepatic	Death
2006	Jackson *et al*.	1	EACA	Preanhepatic	Survived

## DISCUSSION

Other than fibrinolysis, there may be multiple causative factors for excessive bleeding during OLT like thrombocytopenia, dilutional coagulopathy, hypothermia, bleeding due to technical difficulty and inadequate surgical expertise. Antifibrinolytics will decrease bleeding only in cases where it is caused by enhanced fibrinolysis. But they would be harmful in patients with prothrombotic states like Budd-Chiari syndrome, multiorgan transplantation, retransplantation, fulminant liver disease, primary biliary cirrhosis, primary sclerosing cholangitis, renal failure, malignant disease, preexisting thrombotic disease (portal vein thrombosis), and DIC and in paediatric patients. Literature on the use of antifibrinolytic drugs in liver resections is limited.

Among the three antifibrinolytics, aprotinin and TA are widely studied drugs, whereas with EACA, only one RCT is available,[17] which showed no benefit in comparison with placebo. Therefore, until more definitive studies are performed, the role of EACA in OLT will remain ill defined.

Aprotinin and TA have been shown to decrease RBC transfusion requirement in OLT. Aprotinin, but not TA, also reduces intraoperative use of FFP significantly. However, with the FDA warning in 2007 regarding the use of aprotinin,[15] physicians should consider the use of aprotinin in situations where the benefit of reduced blood loss outweighs the potential risks associated with its use.

TA has been shown equally effective as aprotinin in reducing blood loss.[20] When compared to aprotinin it will provide the advantage of being more cost-effective with fewer side effects.

As mentioned earlier, a hyper-coagulable state may also occur in OLT and the risk of thromboembolic complications will increase with use of antifibrinolytics. It is desirable to preoperatively identify the patients who will benefit from an antifibrinolytic drug, thus avoiding extra costs and side effects in patients who do not need the drug. There is no uniform definition of these high-risk cases, but patients with chronic hepatitis, cirrhosis and portal hypertension usually have higher blood loss and are more prone to hyperfibrinolysis. Antifibrinolytics are generally avoided in patients with pre-existing thrombosis, Budd-Chiari syndrome, hepatic artery or portal venous thrombosis.

Another concern is the optimal dosing of the drug. Various dosing schemes have been described in different studies reported so far with no consensus available regarding dosage of any of these three antifibrinolytic drugs.

## CONCLUSION

Reduction in intraoperative bleeding and transfusion requirement with aprotinin and tranexamic acid has been well established in patients undergoing orthotopic liver transplantation. However, patient selection should be on an individual basis to avoid complications. Further large scale and systematic studies are required to draw a firm conclusion about the lowest effective dosages and the risk of thromboembolic complications with antifibrinolytic use.
